# Commentary: A test-retest dataset for assessing long-term reliability of brain morphology and resting-state brain activity

**DOI:** 10.3389/fnins.2017.00085

**Published:** 2017-02-22

**Authors:** João R. Sato, Thomas P. White, Claudinei E. Biazoli

**Affiliations:** ^1^Centre of Mathematics, Computation and Cognition, Universidade Federal do ABCSanto Andre, Brazil; ^2^School of Psychology, University of BirminghamBirmingham, UK

**Keywords:** replicability, brain networks, brain imaging, reproducibility, fingerprints

A transformation toward open neuroscience is ongoing (Milham, 2012), and increases the availability of high-quality, open-access neuroimaging datasets (Poline et al., 2012; Mennes et al., 2013). Consequently, a new set of analytical approaches, including discovery science (Biswal et al., 2010) and focus on individual rather than group-level effects (Finn et al., 2015; Miranda-Dominguez et al., 2014), are increasingly accessible. However, moving to single-subject statistics raises specific concerns that must be addressed. Notable amongst these is the test-retest reliability of fMRI-based metrics (Dubois and Adolphs, 2016). Recently, Huang et al. (2016) provided a test-retest neuroimaging dataset (BNU2), with an inter-scan interval in the order of months, allowing investigation of the temporal reliability of features extracted from rs-fMRI. In the spirit of the data-sharing initiatives, the “Consortium for Reliability and Reproducibility in Functional Connectomics” (CoRR) publicly released this data (Zuo et al., 2014).

Recently, Finn et al. (2015) investigated the existence of functional “connectome fingerprints.” The authors hypothesized that, despite overall similarity in connectivity patterns across subjects, portions of brain connectome variability would be fairly singular to each individual (Mueller et al., 2013; Gordon et al., 2015; Laumann et al., 2015; Xu et al., 2016). This notable study demonstrated that, using only the functional connectivity profile extracted from an fMRI scanning session, it was possible to identify the same subjects from their profiles from a second session a few days later. Interestingly, this hypothesis of a functional connectivity fingerprint was also supported by previous work (Miranda-Dominguez et al., 2014), which additionally showed that such individual signatures exist not only in humans but also in non-human primates.

However, the extent to which connectome profile stability can be generalized to more extended timescales remains largely untested. Furthermore, the vast majority of the functional connectome studies to date focus on timescales of seconds to minutes or years to decades (Poldrack et al., 2015; Huang et al., 2016, but see Xu et al., 2016). We thought that the BNU2 dataset is quite suitable to assess the reliability and stability of connectome fingerprints on an intermediate timescale of months. In fact, the released BNU2 dataset consists of anatomical and functional data from 61 healthy adults (19–23 years old) scanned under a resting-state protocol (eyes closed) in two sessions at an interval of 103–189 days. Further information about scanning parameters, demographical and quality metrics data can be found in Huang et al. (2016).

We preprocessed the data and extracted individual functional connectivity estimates using CONN toolbox version 15.g (Whitfield-Gabrieli and Nieto-Castanon, 2012) with standard MNI152 pipeline and parameters. Conservative options (discarding volumes with displacement >0.5 mm and global-signal *z*-value >3) for scan motion censoring were applied, since motion artifacts are a well-recognized source of error in functional connectivity studies using fMRI. The pairwise bivariate correlations (functional connectivity) among 333 cortical regions-of-interest (ROIs) were obtained using the Gordon et al. (2016) parcellation. Considering the upper triangular values of the individual correlation matrices as the subject connectivity profile, a functional connectome fingerprinting analyses was then carried out. The similarity between the two profiles was then measured with Spearman's correlation coefficient. The within-subject correlation between the two sessions determines the accuracy as it reflects the proportion of subjects correctly identified. Note that the expected accuracy by chance is 1/61 = 1.6%.

We expected to reproduce the original results from connectome fingerprint studies (Miranda-Dominguez et al., 2014; Finn et al., 2015) if the individual profiles are stable over months. In order to do so, we attempted to identify the subjects in the second session based on the profiles similarity to the first session. As a second step, we calculated the intra- and inter-subject similarities between the two sessions for each subject. The inter-subject similarity was calculated by random sampling an individual at the second session. We also sought to investigate how large-scale networks connectivity varies within and between subjects in the timescale of months. Each brain parcel was labeled for the conventional resting-state networks, as provided by the Gordon atlas. Thus, we conducted the two previously described analyses' steps considering all ROIs and each network separately. Based on previous findings of within and between subject variability of network connectivity (Mueller et al., 2013; Miranda-Dominguez et al., 2014; Zuo and Xing, 2014; Chen et al., 2015; Finn et al., 2015; Poldrack et al., 2015), we expected increased discriminability of individuals for heteromodal associative networks.

The results are shown in Figure 1. A high accuracy of 85% for the whole-brain connectivity profile was found. Moreover, accuracies were above 90% for the default mode and the fronto-parietal networks. Interestingly, accuracies for primary sensory and motor networks were lower. It is likely that the ability to uniquely discriminate individuals relies on features with both low within and high between-subject variability over time. For all the networks investigated, we noticed a tendency for higher similarity within subjects than between them. Overall, these results suggest stable connectome fingerprints exist over months and are in agreement with the previously reported inter-individual variability of networks including heteromodal areas. However, caution should be taken when interpreting differences in accuracy between networks as the number and extent of ROIs varies. Since each ROI signal is based on average across voxels, networks with larger parcels may have superior signal-to-noise ratios. Moreover, the number of ROIs may be related to redundancy of information in the connectivity matrices, which would also affect accuracies. Remarkably, the networks which presented the lowest subjects identification accuracies have <8 ROIs.

**Figure 1:**
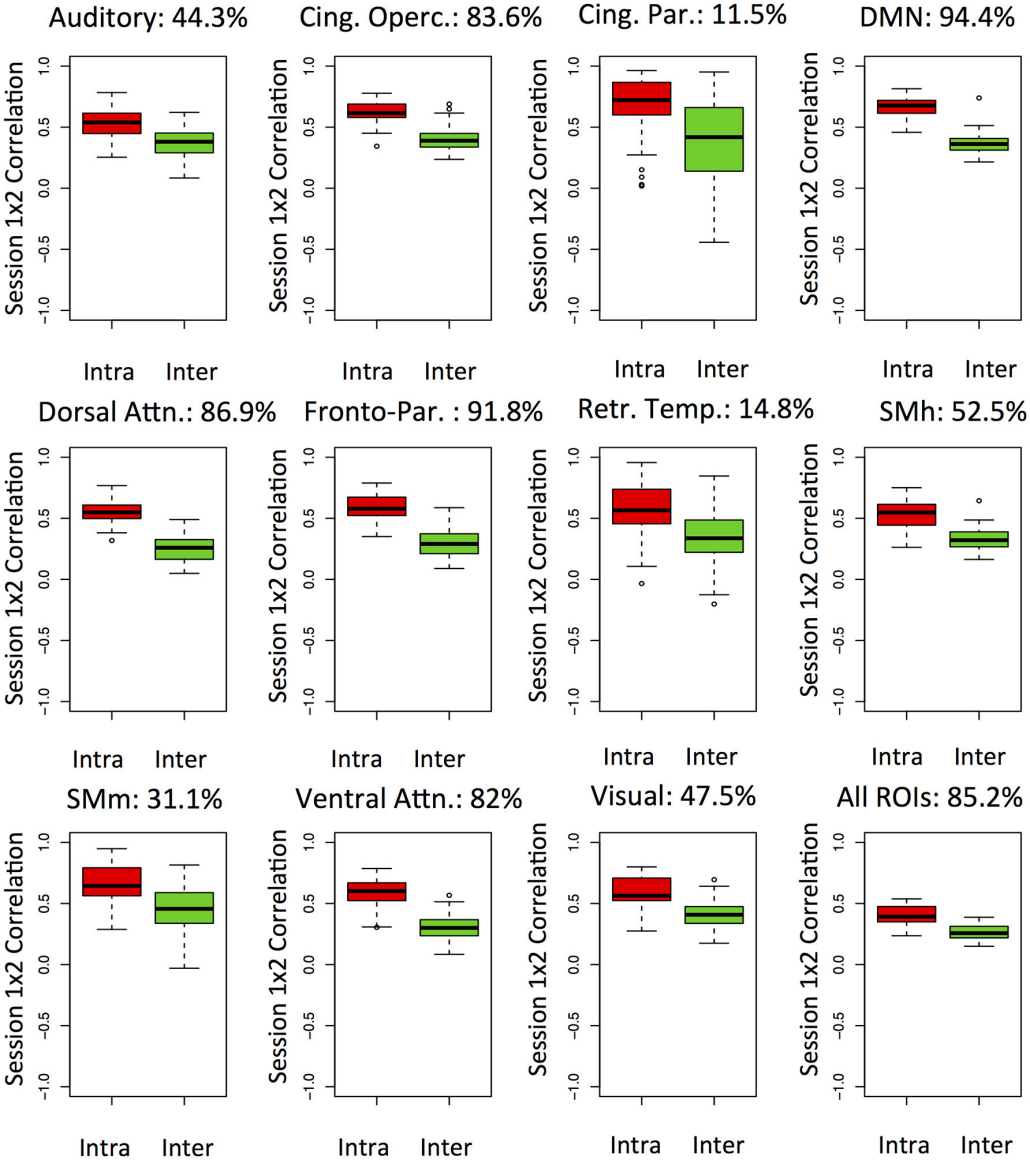
**Functional networks—Box-plots of intra (red) an inter-subject (green) correlations between the first and second resting state fMRI sessions**. The analyses are carried out separated by networks and the connectome fingerprinting accuracy is highlighted as percentage. Cing. Oper., Cingulo-opercular; Cing. Par., Cingulo-parietal; DMN, Default-mode network; Dorsal Attn, Dorsal attention; Fronto-par, Fronto-parietal; Retr. Temp., Retrosplenial-temporal; SMh, somatomotor-hand; SMm, somatomotor-mouth; Ventral Attn, Ventral attention.

Results from individual-based fMRI metrics can be framed by the usual concepts of validity and reliability (Dubois and Adolphs, 2016). However, an inherent issue of the approaches like those proposed here is the extent to which validity and reliability can be disentangled. In other words, it is possible to state that connectome fingerprints are stable over the months, which would constitute a claim for the validity of the underlying neural phenomena. Alternatively, but not mutually exclusively, it is also possible that test-retest reliability varies between subjects and networks. We argue that continuous effort for data-sharing, in the spirit of the CoRR and other initiatives, is of paramount importance as disentangling these factors will ultimately depend on accumulating evidence for the stability of connectome fingerprints across different timescales and with large datasets. Establishing the stability of these measures, in turn, will be essential to investigate true effects of development on the connectomes. Furthermore, adopting comparable acquisition parameters and open and reliable data processing will be necessary to further assure the validity of remarkable findings such as individually unique connectivity profiles.

## Author contributions

JS preprocessed and analyzed the data. All authors wrote the manuscript.

### Conflict of interest statement

The authors declare that the research was conducted in the absence of any commercial or financial relationships that could be construed as a potential conflict of interest.
